# Gait asymmetry and symptom laterality in Parkinson’s disease: two of a kind?

**DOI:** 10.1007/s00415-024-12379-0

**Published:** 2024-04-23

**Authors:** Jana Seuthe, Helen Hermanns, Femke Hulzinga, Nicholas D’Cruz, Günther Deuschl, Pieter Ginis, Alice Nieuwboer, Christian Schlenstedt

**Affiliations:** 1 Institute of Interdisciplinary Exercise Science and Sports Medicine, Medical School Hamburg, Hamburg, Germany; 2 Department of Neurology, Christian-Albrechts-University Kiel, University Hospital Schleswig-Holstein, Kiel, Germany; 3 Neuromotor Rehabilitation Research Group, Department of Rehabilitation Sciences, KU Leuven, Louvain, Belgium

**Keywords:** Parkinson’s disease, Asymmetry, Lateralization, Gait, Turning

## Abstract

**Background:**

The laterality of motor symptoms is considered a key feature of Parkinson’s disease (PD). Here, we investigated whether gait and turning asymmetry coincided with symptom laterality as determined by the MDS-UPRDS part III and whether it was increased compared to healthy controls (HC).

**Methods:**

We analyzed the asymmetry of gait and turning with and without a cognitive dual task (DT) using motion capture systems and wearable sensors in 97 PD patients mostly from Hoehn & Yahr stage II and III and 36 age-matched HC. We also assessed motor symptom asymmetry using the bilateral sub-items of the MDS-UPDRS-III. Finally, we examined the strength of the association between gait asymmetry and symptom laterality.

**Results:**

Participants with PD had increased gait but not more turning asymmetry compared to HC (*p* < 0.05). Only 53.7% of patients had a shorter step length on the more affected body side as determined by the MDS-UPDRS-III. Also, 54% took more time and 29% more steps during turns toward the more affected side. The degree of asymmetry in the different domains did not correlate with each other and was not influenced by DT-load.

**Conclusions:**

We found a striking mismatch between the side and the degree of asymmetry in different motor domains, i.e., in gait, turning, and distal symptom severity in individuals with PD. We speculate that motor execution in different body parts relies on different neural control mechanisms. Our findings warrant further investigation to understand the complexity of gait asymmetry in PD.

**Supplementary Information:**

The online version contains supplementary material available at 10.1007/s00415-024-12379-0.

## Introduction

Asymmetry of motor symptoms is characteristic for Parkinson’s disease (PD) and is particularly observed in the early disease stages. However, asymmetry of symptoms is thought to persist during the entire course of the disease, even if to a lesser degree in the later stages [19]. Previous research suggests that this laterality can be explained by an uneven deficiency of dopamine in the striatum [2], possibly on top of an uneven number of dopaminergic cells in each hemisphere at birth [6]. Furthermore, left–right dominance may play into this asymmetry, as there is some evidence that PD symptoms occur more frequently on the dominant hand-side [34]. Most studies use the bilateral items of the Movement Disorder Society-Unified Parkinson’s Disease Rating Scale part III (MDS-UPDRS III) to determine which body side is more affected by the disease. However, it is unclear whether and how limb asymmetry expresses itself in the gait pattern of individuals with PD. Gait asymmetry is defined as the amount of divergence between the left and right leg for a specific gait parameter. Compared to healthy controls (HC), people with PD show significantly higher gait asymmetry in both the spatial [9, 14] and temporal domain [5, 9, 16, 20] and during various conditions, including treadmill and free-living walking. Gait asymmetry is further increased in individuals with freezing of gait (FOG) and has been identified as a possible trigger of FOG episodes [10, 25]. Turning, a functional gait task with an inherently asymmetric component, is also compromised in people with PD compared to HC [3, 8]. People with PD need more steps for turning than HC and this more so toward the more affected side (measured with the MDS-UPDRS III) [21]. Although a certain degree of motor asymmetry was found in the above-mentioned studies in HC, there is no consensus as to what constitutes an abnormal degree of asymmetry [29, 35].

The question of whether an association exists between symptom laterality and gait- and turning asymmetry is clinically relevant. Recently, rehabilitation studies have aimed to modulate asymmetry using split-belt treadmill paradigms, targeting gait adaptation, turning performance and FOG [7, 13, 24, 30]. In addition, STN-DBS parameters can be adjusted to ameliorate asymmetry to benefit patients. In previous gait asymmetry studies, the label ‘more affected side’ was determined by the MDS-UPDRS III [26, 27, 30], based on the assumption that symptom laterality would correspond with the more affected side of gait. However, Yogev et al. [36] (*n* = 21) and Plotnik et al. [25] (*n* = 36) found that swing time asymmetry and MDS-UPDRS III asymmetry did not correlate. Similarly, Janeh et al. [14] reported that merely one third of participants with FOG took a shorter step on the more affected side (MDS-UPDRS III). A recent study by Plotnik et al. [24] showed that only 5 out of 80 participants had a shorter step length on the more affected body side. Regarding turning, two studies revealed that for self-selected turning direction, people with PD did not show a preference for the more or the less affected side [23, 33]. In terms of turning quality toward the more and less affected side, there are inconsistent findings. Park, et al. [21] found that people with PD without FOG take more steps for turns (360°) toward the less affected side, whereas Freezers took more steps for turns (540°) toward the more affected side. In Freezers, turning direction was not influenced by FOG occurrence; however, FOG started more often at the inner leg of the turn [32].

Given the above-mentioned open questions, we aimed to investigate the association between three aspects of asymmetry, namely (1) gait asymmetry, (2) turning asymmetry, and (3) symptom asymmetry as measured by the MDS-UPDRS III. We hypothesized that the ‘more affected body side’ would also display worse gait performance as expressed by a shorter step length. Given the inconsistent findings in the literature for turning, we used an explorative approach to investigate the associations with turning. We further expected that a higher degree of (absolute) asymmetry in one domain would be associated with a higher degree in another. We also compared gait- and turning asymmetry in people with PD and HC to determine which degree of asymmetry could be considered as ‘abnormal’. Additionally, we compared asymmetry outcomes in subgroups of PD (Freezers vs. Non-Freezers and early vs. advanced PD).

## Methods

This is a cross-sectional sub-analysis of data of the baseline data of two larger trials, conducted at CAU Kiel, Germany and KU Leuven, Belgium (Clinical Trail No: NCT03725215 and NCT04176263). The study designs were described previously [13, 31]. The local ethics committees (Ethical committee of medical faculty at the University Kiel and Ethics Committee Research UZ / KU Leuven) approved the study protocols, the study conforms with the Declaration of Helsinki and participants gave their written informed consent prior to participation.

### Participants

Individuals with PD and healthy controls (HC) were included between February 2018 and May 2021. Inclusion criteria for PD were the diagnosis of idiopathic Parkinson’s disease according to the UK Brain Bank Criteria and the ability to walk at least 5 min unassisted. Exclusion criteria were the presence of (other) neurological diseases, orthopedic or other conditions that influenced gait or balance, contraindicative cardiovascular risk factors or cognitive impairment (Mini-Mental State Examination (MMSE) < 24).

### Testing and outcomes

Assessments were conducted in the ON-state of medication and motor examinations were additionally video-taped to ensure consistency between centers. Descriptive data included age, sex, disease duration and severity (MDS-UPDRS III, Hoehn and Yahr stage), FOG status (freezer or non-freezer) as measured with question 1 of the New FOG Questionnaire, balance performance (Mini Balance Evaluation Systems Test) and cognition (Montreal Cognitive Assessment (MoCA)).

The term ‘symptom asymmetry’ was used for motor symptom asymmetry as measured by the bilateral sub-items of the MDS-UPDRS-III (items 3.3, 3.4, 3.5, 3.6, 3.7, 3.8, 3.15, 3.16, 3.17) and for the lower limb items (items 3.3, 3.7, 3.8, 3.17) separately.

Gait was quantified using 3D-Motion Capture Systems (CAU: Qualisys, KUL: Vicon; sampling frequency 100 Hz) with reflective markers placed at the heel, toe, and ankle of each foot. Gait analysis included straight walking at comfortable pace (10 walking bouts of 10 m, data collection area of 5 m). The following gait variables were computed for each leg separately: step length, swing time and stance time. For turning, participants were asked to perform 360° turns in place in alternating directions, as quickly and safely as possible for one minute. Turning time for each turn was derived from wearable sensor data (APDM Mobility Lab, 128 Hz, placed on the lower back). Additionally, the number of steps for each turn was visually assessed using video material. A step was counted when a foot regained contact to the ground after the entire foot lost contact to the ground. Turning outcomes were obtained for both turn directions separately.

Participants (PD and HC) performed the gait and the turning task without (single task, ST) and with an additional cognitive task (dual task, DT), the latter to possibly tease out higher levels of asymmetry during gait and turning. The DT was an auditory Stroop test, presented and recorded via a wireless headset. Participants heard the words “high” and “low” in either a high or low pitch (congruent and incongruent stimuli) and were asked to name the pitch of the word as fast as possible after each stimulus.

### Definition and quantification of asymmetry

For symptom asymmetry, the more affected side was defined as the side with the higher MDS-UPDRS III sub-score, pooling the bilateral items of the upper and lower limbs (MDS-UPDRS-III_TOTAL_). As an exploratory analysis, we also calculated symptom asymmetry based on the lower limb scores only (MDS-UPDRS-III_LL_). For gait outcomes, the more affected side was defined for several gait outcomes separately being the side with the shorter step length, the shorter stance time and the longer swing time. For turning, the affected side had the longer turning time or the higher number of turning steps.

Asymmetry indexes were calculated for all the above-described outcomes and computed as follows: $\frac{\left(right side-left side\right)}{\left(right side+left side\right)}$.

The asymmetry index entails information about the side of asymmetry and was used to visualize congruency of asymmetry side. Positive values represent asymmetry with the left side being more affected and negative values the right side more affected. The absolute value of the asymmetry indexes was used for the correlation analysis. If a participant obtained a score of 0 on one side for the MDS-UPDRS III, the asymmetry index could not be calculated and, therefore, it was excluded from this analysis (*n* = 3). For the gait outcomes and turning time, we defined a threshold for abnormal asymmetry, which was the mean + 1 standard deviation of the HC data.

### Statistical analysis

Differences between the participants with PD and HC were tested using Mann–Whitney-U Test for ordinal scaled data and Student’s t-test for normally distributed data. Additionally, asymmetries of all outcomes were compared between Freezers and Non-Freezers and between early (H&Y 1 and 2) and advanced (H&Y 3 and 4) PD subgroups, respectively, using students t-Test. The distribution of the less versus the more affected body side between the ST and DT conditions was analyzed with a binomial generalized linear mixed model. The agreement between the more versus the less affected body side in the different domains was investigated descriptively by the number and percentage of participants. Associations between the degree of asymmetry between domains were calculated with Pearson’s correlation coefficient with a confidence level of 95%. Adjustment for multiple testing was performed using Bonferroni correction. The statistical analysis was implemented using RStudio software [28].

## Results

We included *n* = 133 subjects (*n* = 97 individuals with PD (*n* = 67 with FOG) and *n* = 36 HC). Participant characteristics are shown in Tables 1, 2 comparing PD and HC and in Table 3 comparing Freezers and Non-freezers. Individuals with PD and HC did not differ significantly regarding age and sex (*p* > 0.05).

**Table 1:** Participant characteristics (*n* = 133)

	PD (*n* = 97)	HC (*n* = 36)	*p*-values
Age (yrs)	66.86 (10.03)	69.64 (6.57)	0.169
Sex (f/m)	28/69	16/20	0.136
MoCA	25.31 (2.99)	27.11(2.61)	< 0.001*
Mini-BESTest	21.91 (4.84)	25.33(2.35)	< 0.001*
DD (yrs)	9.86 (6.8)	–	–
H&Y (1/2/3/4)	2 (1–4) (1/61/29/6)	–	–
MDS-UPDRS III	34.4 (14.16)	–	–
NFOG-Q^#^ (*n* = 64)	16.05 (5.73)	–	–
Age (yrs)	66.86 (10.03)	69.64 (6.57)	0.169
Sex (f/m)	28/69	16/20	0.136
MoCA	25.31 (2.99)	27.11(2.61)	< 0.001*
Mini-BESTest	21.91 (4.84)	25.33(2.35)	< 0.001*
DD (yrs)	9.86 (6.8)	–	–
H&Y (1/2/3/4)	2 (1–4) (1/61/29/6)	–	–
MDS-UPDRS III	34.4 (14.16)	–	–
NFOG-Q^#^ (*n* = 64)	16.05 (5.73)	–	–

*MoCA* Montreal Cognitive Assessment, *DD* disease duration, *H&Y* Hoehn and Yahr stage, *MDS-UPDRS III* movement disorder society-unified Parkinson’s disease rating scale part III; *NFOG-Q* new freezing of gait questionnaire; Data on disease duration, MDS-UPDRS-III and MoCA are for *n* = 96; # for *n* = 64 (only individuals with FOG)

*Statistically significant (*p* < 0.05). Presented are mean (sd)

**Table 2:** Comparison of gait- and turning asymmetries (PD vs. HC)

	PD (*n* = 97)	HC (*n* = 36)	*p*-values
Step length asymmetry			
Mean (sd)	0.029 (0.031)	0.016 (0.010)	< 0.001*
Median (1st–3rd quartiles)	0.022 (0.007–0.040)	0.012 (0.007–0.022)	–
Swing time asymmetry			
Mean (sd)	0.011 (0.010)	0.006 (0.006)	< 0.001*
Median (1st–3rd quartiles)	0.008 (0.003–0.015)	0.005 (0.001–0.010)	–
Stance time asymmetry			
Mean (sd)	0.008 (0.008)	0.005 (0.004)	0.003*
Median (1st–3rd quartiles)	0.006 (0.003–0.011)	0.005 (0.002–0.008)	–
Turning time asymmetry			
Mean (sd)	0.034 (0.050)	0.024 (0.018)	0.077
Median (1st–3rd quartiles)	0.022 (0.011–0.047)	0.023 (0.009–0.035)	–
Number of steps asymmetry			
Mean (sd)	0.038 (0.040)	–	–
Median (1st–3rd quartiles)	0.026 (0.015–0.052)	–	–

Results of the single task condition are presented

*Significant (*p* < 0.05)

**Table 3:** Congruency between symptom- and gait asymmetry

		*n*	More affected side *n* (%)	Less affected side *n* (%)	No asymmetry in MDS-UPDRS-III *n* (%)	Missing value for MDS-UPDRS-III *n* (%)
Comparison with MDS-UPDRS-III_TOTAL_						
Shorter step length	All	97	59 (60.82)	29 (29.9)	8 (8.25)	1 (1.03)
	>|mean + sd|	41	22 (53.66)	14 (34.15)	4 (9.76)	1 (2.44)
Shorter stance time	All	96†	59 (61.46)	28 (29.17)	8 (8.25)	1 (1.04)
	>|mean + sd|	36	22 (61.11)	12 (33.33)	2 (5.55)	0 (0)
Longer swing time	All	97	56 (57.73)	32 (32.99)	8 (8.25)	1 (1.03)
	>|mean + sd|	32	23 (71.88)	7 (21.88)	2 (6.25)	0 (0)
Comparison with MDS-UPDRS-III_LL_						
Shorter step length	All	97	49 (50.52)	28 (28.87)	19 (19.58)	1 (1.03)
	>|mean + sd|	41	24 (58.54)	9 (21.95)	7 (17.07)	1 (2.44)
Shorter stance time	All	96†	51 (53.13)	25 (26.04)	19 (19.79)	1 (1.04)
	>|mean + sd|	36	19 (52.77)	7 (19.44)	10 (27.77)	0 (0)
Longer swing time	All	97	47 (48.45)	30 (30.93)	19 (19.58)	1 (1.03)
	>|mean + sd|	32	16 (50)	6 (18.75)	10 (31.25)	0 (0)

Congruency and incongruency with our hypothesis are indicated with green and red shading, respectively

^†^One participant with no asymmetry due to equal stance time on both sides

The degree of gait and turning asymmetry and the distribution of the lesser versus the more affected side did not significantly differ between the ST and the DT condition (Supplementary Table 1). In the following, only the data of the ST condition is presented. Data regarding the DT condition can be found in the supplementary material (Tables 2, 3, 4).

**Table 4:** Congruency between symptom- and turning asymmetry

		*n*	More affected side is the outer leg *n* (%)	More affected side is the inner leg *n* (%)	No asymmetry in MDS-UPDRS-III *n* (%)	No asymmetry while turning *n* (%)	Missing value in MDS-UPDRS-III *n* (%)	Missing value in turning *n* (%)	Missing value in turning and no asymmetry in MDS-UPDRS-III *n* (%)
Comparison with MDS-UPDRS-III_TOTAL_									
Longer turning time	All	97	33 (34.02)	52 (53.61)	8 (8.25)	0 (0)	1 (1.03)	3 (3.09)	0 (0)
	>|mean + sd|	28	13 (46.43)	11 (39.29)	4 (14.28)	–	–	–	–
More steps	All	97	43 (44.33)	28 (28.87)	5 (5.15)	2 (2.06)	1 (1.03)	15 (15.46)	3 (3.09)
Comparison with MDS-UPDRS-III_LL_									
Longer turning time	All	97	34 (35.05)	39 (40.21)	20 (20.62)	0 (0)	1 (1.03)	3 (3.09)	0 (0)
	>|mean + sd|	28	12 (42.86)	9 (32.14)	7 (25.00)	–	–	–	–
More steps	All	97	33 (34.02)	25 (25.77)	18 (18.56)	2 (2.06)	1 (1.03)	16 (16.49)	2 (2.06)

Congruency and incongruency with our hypothesis are indicated with green and red shading, respectively

### Asymmetry in symptom laterality (MDS-UPDRS-III)

Eighty-eight (90.7%) of the 97 individuals with PD presented with symptom asymmetry according to the MDS-UPDRS-III_TOTAL_. Fifty-two (59,0%) had a more affected left side and 36 (40.9%) had a more affected right side (and 9 had no asymmetry). Seventy-six (87.4%) participants presented with disease asymmetry based on the scores of the lower extremity items (MDS-UPDRS-III_LL_).

### Asymmetry in people with PD versus HC

**Fig. 1:**
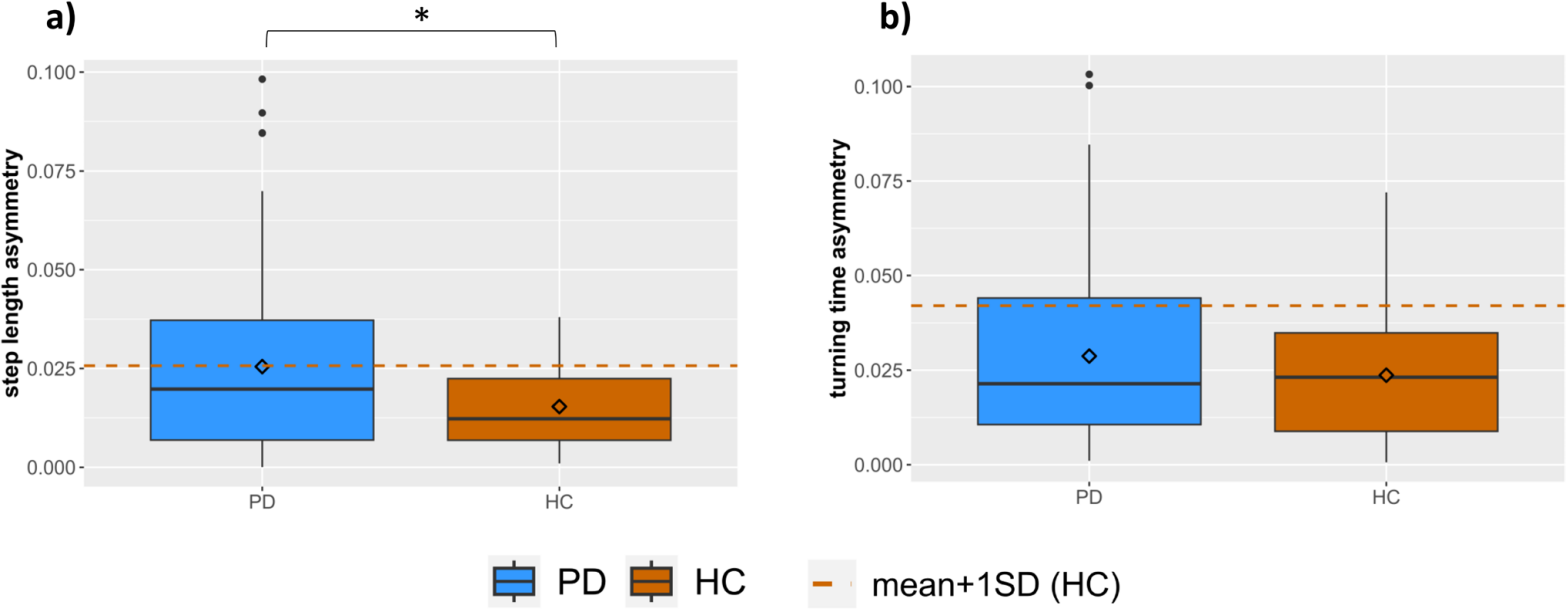
Asymmetry of step length and turning time during single task conditions. Dashed lines represent the mean + 1 standard deviation for HC. *Statistically significant difference

Step length asymmetry differed significantly between people with PD and HC (*p* < 0.001, Table 2), whereby PD had more asymmetry. Similarly, temporal gait asymmetry for swing- (*p* < 0.001) and stance time (*p* = 0.003) was greater in PD. However, turning time asymmetry was not significantly different between PD and HC (*p* = 0.077). Figure 1 shows the step length asymmetry and turning time asymmetry values of PD and HC. The number of people with PD who had step length asymmetry and turning time asymmetry above the abnormal threshold (mean + 1 SD of HC values) was *n* = 41 (42.3%) and *n* = 28 (28.9%), and for HC *n* = 7 (19.4%) and *n* = 4 (11.1%), respectively.

### Asymmetry in Freezers versus Non-Freezers

Freezers did not differ significantly from Non-Freezers regarding age, sex and MoCA but performed worse for the Mini-BESTest and MDS-UPDRS III and had longer disease duration (*p* < 0.05, Table 5 supplemental material online). Gait asymmetry did not differ significantly between Freezers and Non-Freezers (step length asymmetry: *p* = 0.200, swing time asymmetry: *p* = 0.127, stance time asymmetry: *p* = 0.175). Similarly, turning time asymmetry was not significantly different between the groups (*p* = 0.102). However, turning step asymmetry was significantly higher in the Freezers compared to Non-Freezers (*p* = 0.010). For asymmetry of motor symptoms, the Non-Freezers showed higher asymmetry values (*p* = 0.029). For details see Table 6 in the supplemental material online.

**Table 5:** Congruency of gait- and turning asymmetry

		*n*	Outer leg with shorter step length *n* (%)	Inner leg with shorter step length *n* (%)	No asymmetry while turning *n* (%)	Missing value for turning *n* (%)
Longer turning time	All	97	41 (42.27)	53 (54.64)	0 (0)	3 (3.1)
	>|mean + sd|	41	15 (36.59)	24 (58.54)	0 (0)	2 (4.88)
More steps	all	97	35 (36.08)	41 (42.27)	21 (10.31)	0 (0)
	>|mean + sd|	41	23 (56.1)	12 (29.27)	0 (0)	6 (14.63)

Congruency and incongruency with our hypothesis are indicated with green and red shading, respectively

### Asymmetry in early versus advanced PD

When comparing the early versus advanced participants with PD, we did not find any significant differences in gait, turning or symptom asymmetries (*p* < 0.05, for details see Table 7 in the supplemental material online).

### Congruency between asymmetry domains

#### Symptom and gait asymmetry

**Fig. 2:**
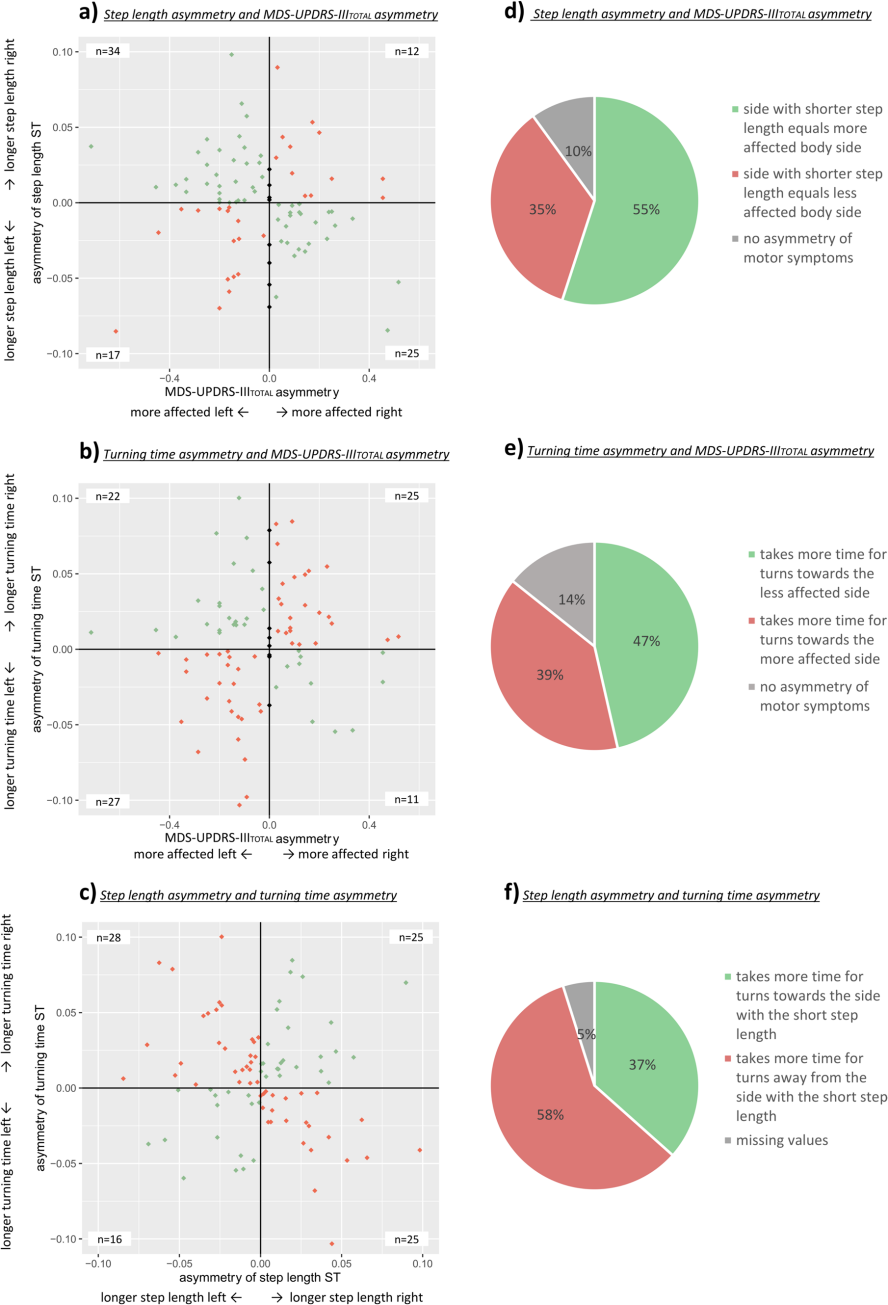
**A** asymmetry of step length and MDS-UPDRS-III_TOTAL_ asymmetry, *n* = 94. **B** asymmetry of turning time and MDS-UPDRS-III_TOTAL_ asymmetry, *n* = 95. **C** asymmetry of turning time and asymmetry of step length, *n* = 93. green points: participants with consistent asymmetric sides, red points: participants with no consistent asymmetric sides, gray points: represent participants with no asymmetry in MDS-UPDRS-III_TOTAL_; **D**, **E** and **F** represent percentages based on the ‘pathological’ sample according to the cut-off (**D** and **F**) *n* = 41; **E** *n* = 28). For **F** the side participants are turning toward is the inner leg; the side participants are turning away from is the outer leg is the *ST* single task, *LL* lower limb

Considering only the 41 (step length), 36 (stance time), 32 (swing time) participants with abnormal gait asymmetry, 53,7% showed a shorter step length, 61,1% a shorter stance time and 71,9% a longer swing time on the more affected side according to the MDS-UPDRS-III_TOTAL_. When considering only the MDS-UPDRS-III_LL_, the agreement tended to decrease, with only about 50% with congruent asymmetry (Table 5). The agreement between asymmetry domains is visualized in Fig. 2A, where the green points represent the participants with congruent asymmetries and the red dots indicate the discrepancies. The agreement for the subgroup with abnormal gait asymmetry is presented in Fig. 2D, showing that the majority of participants show congruent gait and symptom asymmetry.

#### Symptom- and turning asymmetry

A higher number of patients (54%) needed more time for turns when turning toward the more affected side. When considering only the MDS-UPDRS-III_LL_, the results did not change substantially. The results can be found in detail in Table 4. Figure 2B illustrates the agreement, where the green points represent the participants with congruent asymmetries and the red dots indicate the discrepancies. When looking at the subgroup with abnormal turning asymmetry, relatively more people took longer for turns toward the less affected side (hence, when the more affected side is the outer leg during the turn, see Fig. 2E).

#### Gait- and turning asymmetry

Forty-two percent of participants with PD had a longer turning time when the side with the shorter step length was at the outer side of the turn. Yet, 55% had a longer turning time when the side with the shorter step length was at the inner side while turning (see Fig. 2C). Regarding the number of steps, 29% of participants needed more steps when the leg with the shorter step length was at the inner side of the turn. Detailed results are presented in Table 5.

When we adjusted the analysis, by looking at the ‘abnormal asymmetry’ values only, the results changed slightly. Figure 2F shows that only 37 percent of participants took more time for turns, when the leg with the shorter step length was the outer leg during the turn, whereas the absolute majority of participants (58%) took more time when the side with the longer step length was the outer leg during the turn.

### Correlations between the asymmetry indexes of gait, turning, and overall symptoms

There were no significant correlations between symptom laterality (MDS-UPDRS-III_TOTAL_ and MDS-UPDRS-III_LL_) and the gait asymmetry index or the turning asymmetry index. The results of the correlation analysis are presented in Table 8 in the supplementary material.

### Sub-analysis of Freezers

The sub-analysis of congruency between asymmetry domains in Freezers also showed inconsistent results. Depending on the outcome congruency between gait asymmetry and symptom laterality ranged between 40 and 71%. For turning asymmetry and symptom asymmetry, we found more participants, taking more time for turns, when turning toward the more affected side (for details, see Tables 9, 10, 11 in supplemental material online).

## Discussion

This study compared different domains of asymmetry and their associations in individuals with PD and HC. We provided reference data for gait and turning asymmetries for HC, and showed that people with PD had higher asymmetry during gait, but not during turning. Forty-two percent and 29% of individuals with PD were classified as having abnormal step length or turning asymmetries based on our cut-off. Most importantly, and in contrast to our hypothesis we found a relatively high number of participants with incongruent gait and turning asymmetry in relation to their symptom laterality. More specifically, in about one third the side with greater symptom severity was the side with the larger step length during walking. For turning, about half of the participants had a longer turn duration, when the inner leg was more affected. Focusing only on the MDS-UPDRS-III_LL_ scores did not change the proportions of these mismatches. In participants with FOG, we also found large incongruences.

Our findings suggest that gait and turning asymmetry is most likely caused by complex mechanisms, which include but are not limited to symptom distribution in the limbs. We speculate that the degree of deterioration of postural control may also contribute to the asymmetry incongruences. However, as we found that turning asymmetry had no association with gait asymmetry, the involvement of postural control may only partly explain the asymmetry mismatch. Although gait and turning both involve bipedal stepping and forward locomotion, the two motor tasks are different in several aspects. Compared to gait, turning involves gaze rotation and more complex coordination between the head, trunk, pelvis and feet [4, 11]. Turning also involves more interlimb coordination, multisensory integration with a greater vestibular component than gait [1, 15] and asymmetrical modifications of the locomotor patterns [11, 22]. Turning problems can occur early in the disease while gait disorders usually occur later [12]. There is some evidence that during turning, people with PD show reduced prefrontal cortex activation compared to regular walking [17], underlining the different cortical demands of these two tasks.

Comparing the degree of asymmetry by associating the index values in the different motor domains, no significant correlations were found after adjusting for multiple testing. This underscores that increased asymmetry in gait or turning is not consistently explained by symptom laterality. The differences between distal and axial asymmetry may be attributed to the nature of the different motor tasks. MDS-UPDRS-III items often require the fast production of repetitive movements. These tasks are less related to functional motor tasks such as gait and turning which involve postural control and are usually performed at comfortable speed. However, when testing DT-gait the same results were found as in ST-conditions. Symptom asymmetry was larger in the Non-Freezer group. One explanation might be the significantly higher disease duration in Freezers (despite similar symptom severity). Despite some evidence for a decrease in asymmetry with advanced disease duration [18], we did not confirm higher asymmetry scores in early versus advanced patients in this cohort.

### Clinical implications

Our findings show a clear mismatch between asymmetry of gait, turning and overall symptoms. In the past, therapy modalities were often chosen solely based on symptom laterality. The findings of our study suggest to at least consider differences in distribution of asymmetrical impairment in different domains. If treatment is aimed at a single domain, we recommend to base choices specifically on the aim of the treatment, e.g., if the aim is to alleviate step length asymmetry, step length laterality should guide therapy. However, for treatments that address a cluster of symptoms, the MDS-UPDRS III probably remains the best option.

### Limitations

This work has some limitations. First, we encountered the issue that not all participants in this study presented an asymmetric MDS-UPDRS III score and even less so regarding the lower limb items, reducing the number of included subjects for each research question. Importantly, we had no pathological gold standard measure of asymmetry, such as an initial DAT-scan. Future work should include neuroimaging parameters indicative of pathological asymmetry. Also, participants were assessed ON-medication, which may have influenced symptoms in different domains differentially. Furthermore, to our knowledge, there is no previous work about cut-off values which define asymmetry. Hence, we used the mean + 1SD as an arbitrary classification based on a relatively small HC group. The definition and validity of what constitutes ‘abnormal asymmetry’, needs further verification in a different cohort exploring various cut-offs.

## Conclusions

We found a significant mismatch between asymmetry of gait and turning and the laterality of symptom severity in the limbs as tested by the MDS-UPDRS-III items. Future studies should therefore carefully consider the specific motor tests used to determine motor asymmetry in different domains. Our findings also suggest that motor asymmetry is a complex construct which needs further investigation.

### Supplementary Information

Below is the link to the electronic supplementary material.

Supplementary file1 (DOCX 49 kb)

## Data Availability

The data that support the findings of this study are not publicly available due to restrictions of the local ethics committee, but can be received from the corresponding author upon reasonable request.
