# Supplementary material for: Gait asymmetry and symptom laterality in Parkinson’s disease: two of a kind?
Source: J Neurol. 2024 Apr 23;271(7):4373–82. doi: 10.1007/s00415-024-12379-0 (PMC11233399; doi:10.1007/s00415-024-12379-0)
Supplement: Supplementary file 1 — Supplementary file1 (DOCX 49 kb) [file 415_2024_12379_MOESM1_ESM.docx]

**Supplementary material**

Manuscript title: Gait asymmetry and symptom laterality in Parkinson’s disease - two of a kind?

Journal name: Journal of Neurology

Jana Seuthe^1,2^*, Helen Hermanns^2^*, Femke Hulzinga^3^, Nicholas D’Cruz^3^, Günther Deuschl^2^, Pieter Ginis^3^, Alice Nieuwboer^3^, Christian Schlenstedt^1,2^

^1^Institute of Interdisciplinary Exercise Science and Sports Medicine, Department Performance, Neuroscience, Therapy and Health, Medical School Hamburg, Germany

^2^Christian-Albrechts-University Kiel, University Hospital Schleswig-Holstein, Department of Neurology, Kiel, Germany

^3^ KU Leuven, Neuromotor Rehabilitation Research Group, Department of Rehabilitation Sciences, Leuven, Belgium

*authors contributed equally

Corresponding author: Dr. Jana Seuthe

Email: jana.seuthe@medicalschool-hamburg.de

**Table 1**. Binomial generalized mixed model results for difference of distribution of the more affected side (left or right) for gait outcomes between single task and dual task conditions

|  | z-value | p-value |
| --- | --- | --- |
| Step length | 0.433 | 0.665 |
| Stance time | 0.000 | 1.000 |
| Swing time | 0.287 | 0.774 |
| Turning steps | 1.860 | 0.063 |
| Turning time | -0.382 | 0.703 |

***Table 2.*** *Comparison MDS-UPDRS-III score (all items and subitems for lower extremity) and gait parameters (dual task).*

|  |  | | n | more affected side  n (%) | less affected side  n (%) | no asymmetry in  MDS-UPDRS-III  n (%) | missing value for  MDS-UPDRS-III  n (%) |
| --- | --- | --- | --- | --- | --- | --- | --- |
| Comparison with  MDS-UPDRS-III | **shorter step length** | all | 97 | 57 (58.76) | 31 (31.96) | 8 (8.25) | 1 (1.03) |
|  |  | >\|mean + sd\| | 45 | 27 (60) | 15 (33.33) | 2 (4.44) | 1 (2.22) |
|  | **shorter stance time** | all | 97 | 56 (57.73) | 32 (32.99) | 8 (8.25) | 1 (1.03) |
|  |  | >\|mean + sd\| | 27 | 20 (74.07) | 6 (22.22) | 1 (3.7) | 0 (0) |
|  | **longer swing time** | all | 97 | 57 (58.76) | 31 (31.96) | 8 (8.25) | 1 (1.03) |
|  |  | >\|mean + sd\| | 30 | 22 (73.33) | 6 (20) | 2 (6.66) | 0 (0) |
| Comparison with  MDS-UPDRS-III subitems for lower extremity | **shorter step length** | all | 97 | 48 (49.48) | 29 (29.9) | 19 (19.59) | 1 (1.03) |
|  |  | >\|mean + sd\| | 45 | 22 (48.88) | 13 (28.88) | 9 (20) | 1 (2.22) |
|  | **shorter stance time** | all | 97 | 46 (47.42) | 31 (31.96) | 19 (19.59) | 1 (1.03) |
|  |  | >\|mean + sd\| | 27 | 15 (55.55) | 6 (22.22) | 6 (22.22) | 0 (0) |
|  | **longer swing time** | all | 97 | 48 (49.48) | 29 (29.9) | 19 (19.59) | 1 (1.03) |
|  |  | >\|mean + sd\| | 30 | 17 (56.66) | 6 (20) | 7 (23.33) | 0 (0) |

Note. Congruency and incongruency with our hypothesis are indicated with green and red shading respectively.

***Table 3.*** *Comparison MDS-UPDRS-III score (all items and subitems for lower extremity) and turning parameters (dual task).*

|  |  | **n** | **more affected side is the**  **outer leg**  **n (%)** | **more affected side is the**  **inner leg**  **n (%)** | **no asymmetry in**  **MDS-UPDRS-III**  **n (%)** | **no asymmetry while turning**  **n (%)** | **missing value in MDS-UPDRS-III**  **n (%)** | **missing value in turning**  **n (%)** | **missing value in turning and no asymmetry in MDS-UPDRS-III**  **n (%)** |
| --- | --- | --- | --- | --- | --- | --- | --- | --- | --- |
| **Comparison with**  **MDS-UPDRS-III** | **longer turning time** | 97 | 43 (44.33) | 42 (43.3) | 7 (7.23) | 0 (0) | 1 (1.03) | 3 (3.09) | 1 (1.03) |
|  | **more steps** | 97 | 35 (36.08) | 35 (36.08) | 5 (5.15) | 2 (2.06) | 1 (1.03) | 16 (16.49) | 3 (3.09) |
| **Comparison with**  **MDS-UPDRS-III subitems for lower extremity** | **longer turning time** | 97 | 41 (42.27) | 32 (32.99) | 19 (19.59) | 0 (0) | 0 (0) | 1 (1.03) | 3 (3.09) |
|  | **more steps** | 97 | 27 (27.84) | 32 (32.99) | 16 (16.49) | 1 (1.03) | 1 (1.03) | 1 (1.03) | 16 (16.49) |

Note. Congruency and incongruency with our hypothesis are indicated with green and red shading respectively.

***Table 4.*** *Comparison gait parameters and turning parameters (dual task).*

|  |  | n | **outer leg with shorter step length**  n (%) | **inner leg with shorter step length**  n (%) | **no asymmetry while turning**  n (%) | **missing value for turning**  n (%) |
| --- | --- | --- | --- | --- | --- | --- |
| **longer turning time** | all | 97 | 46 (47.42) | 48 (49.48) | 0 (0) | 3 (3.1) |
|  | >\|mean + sd\| | 45 | 20 (44.44) | 25 (55.55) | 0 (0) | 0 (0) |
| **more steps** | all | 97 | 39 (40.21) | 37 (38.14) | 21 (10.31) | 0 (0) |
|  | >\|mean + sd\| | 45 | 17 (37.77) | 18 (40) | 9 (20) | 1 (2.22) |

Note. Congruency and incongruency with our hypothesis are indicated with green and red shading respectively.

|  |  | **Freezers (n=67)** | **Non-Freezers (n=30)** | **p-values** |
| --- | --- | --- | --- | --- |
| Participant characteristics | Age (yrs) | 67.69 (9.64) | 65.10 (10.78) | 0.333 |
|  | Sex (f/m) | 18/49 | 10/20 | 0.684 |
|  | MoCA | 24.94 (3.23) | 26.17 (2.21) | 0.095 |
|  | Mini-BESTest | 21.18 (4.92) | 23.53 (4.30) | 0.008 |
|  | DD (yrs) | 11.95 (6.87) | 5.27 (3.70) | <0.001 |
|  | H&Y  (1/2/3/4) | 1/34/26/6 | 0/27/3/0 | 0.003 |
|  | MDS-UPDRS III | 35.61 (14.82) | 31.87 (12.29) | 0.266 |

***Table 5****. Clinical characteristics of participants (Freezers vs. Non-Freezers).*

***Table 6****. Comparison of asymmetries between freezers and non-freezers.*

|  |  | **Freezers (n=67)** | **Non-Freezers (n=30)** | **p-values** |
| --- | --- | --- | --- | --- |
| step length asymmetry | Mean (sd) | 0.031 (0.034) | 0.024 (0.025) | 0.200 |
|  | Median (1^st^ - 3^rd^ quartile) | 0.025 (0.009-0.043) | 0.014 (0.006-0.107) |  |
| swing time asymmetry | Mean (sd) | 0.012 (0.011) | 0.009 (0.008) | 0.127 |
|  | Median (1^st^ - 3^rd^ quartile) | 0.010 (0.004-0.015) | 0.006 (0.002-0.013) |  |
| stance time asymmetry | Mean (sd) | 0.009 (0.009) | 0.007 (0.006) | 0.175 |
|  | Median (1^st^ - 3^rd^ quartile) | 0.008 (0.003-0.012) | 0.005 (0.002-0.010) |  |
| turning time asymmetry | Mean (sd) | 0.039 (0.059) | 0.025 (0.021) | 0.102 |
|  | Median (1^st^ - 3^rd^ quartile) | 0.028 (0.009-0.050) | 0.019 (0.011-0.029) |  |
| number of steps (during turns) asymmetry | Mean (sd) | 0.046 (0.045) | 0.025 (0.026) | 0.010* |
|  | Median (1^st^ - 3^rd^ quartile) | 0.045 (0.019-0.059) | 0.021(0.010-0.031) |  |
| MDS-UPDRS-III asymmetry | Mean (sd) | 0.154 (0.159) | 0.246 (0.194) | 0.029* |
|  | Median (1^st^ - 3^rd^ quartile) | 0.123 (0.061-0.200) | 0.193 (0.125-0.321) |  |

|  |  |  | **Less affected PD (n=62)** | **More affected PD (n=35)** | **p-values** |
| --- | --- | --- | --- | --- | --- |
| Measures of asymmetry | step length | Mean (sd) | 0.024 (0.020) | 0.038 (0.044) | 0.076 |
|  |  | Median (1^st^ - 3^rd^ quartile) | 0.019 (0.008-0.035) | 0.024 (0.007-0.057) |  |
|  | swing time | Mean (sd) | 0.010 (0.009) | 0.012 (0.012) | 0.317 |
|  |  | Median (1^st^ - 3^rd^ quartile) | 0.008 (0.002-0.013) | 0.010 (0.005-0.015) |  |
|  | stance time | Mean (sd) | 0.008 (0.007) | 0.009 (0.009) | 0.673 |
|  |  | Median (1^st^ – 3^rd^ quartile) | 0.006 (0.003-0.011) | 0.007 (0.003-0.012) |  |
|  | turning time | Mean (sd) | 0.028 (0.023) | 0.046 (0.078) | 0.216 |
|  |  | Median (1^st^ – 3^rd^ quartile) | 0.021 (0.011-0.045) | 0.026 (0.012-0.048) |  |
|  | number of steps (during turns) | Mean (sd) | 0.038 (0.045) | 0.039 (0.027) | 0.916 |
|  |  | Median (1^st^ – 3^rd^ quartile) | 0.026 (0.015-0.046) | 0.030 (0.019-0.056) |  |
|  | MDS-UPDRS-III | Mean (sd) | 0.198 (0.160) | 0.155 (0.198) | 0.279 |
|  |  | Median (1^st^ - 3^rd^ quartile) | 0.164 (0.091-0.250) | 0.119 (0.036-0.161) |  |

***Table 7.*** *Comparison of asymmetries between less- (H&Y 1-2) and more affected (H&Y 3-4) participants with PD.*

***Table 8.*** *The Association between gait and turning asymmetry (absolute) and symptom laterality.*

|  | | **n** | **MDS-UPDRS-III_total_ asymmetry** | **n** | **MDS-UPDRS-III_LL_ asymmetry** |
| --- | --- | --- | --- | --- | --- |
|  | |  | **single task** |  | |
| **step length** | | 95 | r = -0.017, p = 1 | 92 | r = - 0.076, p = 1 |
| **stance time** | | 95 | r = 0.056, p = 1 | 92 | r = 0.048, p = 1 |
| **swing time** | | 95 | r = 0.096, p = 1 | 92 | r = 0.045, p = 1 |
| **turning time** | | 95 | r = - 0.046, p = 1 | 92 | r = 0.017, p = 1 |
| **number of steps** | | 95 | r = - 0.237, p = 0.38 | 92 | r = - 0.049, p = 1 |
|  |  |  | **dual task** |  |  |
| **step length** | | 95 | r = 0.05, p = 1 | 92 | r = - 0.04, p = 1 |
| **stance time** | | 95 | r = 0.133, p = 1 | 92 | r = 0.072, p = 1 |
| **swing time** | | 95 | r = 0.172, p = 0.96 | 92 | r = 0.071, p = 1 |
| **turning time** | | 95 | r = - 0.186, p = 0.78 | 92 | r = - 0.187, p = 0.81 |
| **number of steps** | | 95 | r = - 0.236, p = 0.4 | 92 | r = - 0.054, p = 1 |

Note. r=Pearson´s correlation coefficients. P-values were adjusted with bonferroni-correction for multiple testing.

***Table 9.*** *Comparison MDS-UPDRS-III score (all items and subitems for lower extremity) and gait parameters (in* ***Freezers****)*

|  |  | | n | more affected side  n (%) | less affected side  n (%) | no asymmetry in  MDS-UPDRS-III  n (%) | missing value for  MDS-UPDRS-III  n (%) |
| --- | --- | --- | --- | --- | --- | --- | --- |
| Comparison with  MDS-UPDRS-III | **shorter step length** | all | 67 | 41 (61.19) | 17 (25.37) | 8 (11.94) | 1 (1.49) |
|  |  | >\|mean + sd\| | 32 | 19 (59.38) | 8 (25.00) | 5 (15.62) | 0 (0.00) |
|  | **shorter stance time** | all | 67 | 34 (50.75) | 24 (35.82) | 8 (11.94) | 1 (1.49) |
|  |  | >\|mean + sd\| | 7 | 5 (71.43) | 1 (14.28) | 1 (14.28) | 0 (0.00) |
|  | **longer swing time** | all | 67 | 36 (54.73) | 22 (32.84) | 8 (11.94) | 1 (1.49) |
|  |  | >\|mean + sd\| | 25 | 15 (60.00) | 8 (32.00) | 2 (8.00) | 0 (0.00) |
| Comparison with  MDS-UPDRS-III subitems for lower extremity | **shorter step length** | all | 67 | 32 (47.76) | 20 (29.85) | 14 (20.99) | 1 (1.49) |
|  |  | >\|mean + sd\| | 32 | 18 (56.25) | 7 (21.88) | 7 (21.88) | 0 (0.00) |
|  | **shorter stance time** | all | 67 | 31 (46.27) | 21 (31.34) | 14 (20.99) | 1 (1.49) |
|  |  | >\|mean + sd\| | 7 | 4 (57.14) | 1 (14.29) | 2 (28.57) | 0 (0.00) |
|  | **longer swing time** | all | 67 | 34 (50.75) | 18 (26.87) | 14 (20.99) | 1 (1.49) |
|  |  | >\|mean + sd\| | 25 | 10 (40.00) | 7 (28.00) | 8 (32.00) | 0 (0.00) |

Note. Congruency and incongruency with our hypothesis are indicated with green and red shading respectively.

***Table 10.*** *Comparison MDS-UPDRS-III score (all items and subitems for lower extremity) and turning parameters (in* ***Freezers****)*

|  |  |  | **n** | **more affected side is the**  **outer leg**  **n (%)** | **more affected side is the**  **inner leg**  **n (%)** | **no asymmetry in**  **MDS-UPDRS-III**  **n (%)** | **no asymmetry while turning**  **n (%)** | **missing value in MDS-UPDRS-III**  **n (%)** | **missing value in turning**  **n (%)** | **missing value in turning and no asymmetry in MDS-UPDRS-III**  **n (%)** |
| --- | --- | --- | --- | --- | --- | --- | --- | --- | --- | --- |
| **Comparison with**  **MDS-UPDRS-III** | **longer turning time** | all | 67 | 19 (28.36) | 36 (53.73) | 8 (11.94) | 0 (0.00) | 1 (1.49) | 3 (4.48) | 0 (0.00) |
|  |  | >\|mean + sd\| | 25 | 5 (20.00) | 15 (60.00) | 2 (8.00) | 0 (0.00) | 0 (0.00) | 3 (12.00) | 0 (0.00) |
|  | **more steps** | all | 67 | 31 (46.27) | 11 (16.42) | 6 (8.96) | 1 (.149) | 1 (1.49) | 17 (25.37) | 0 (0.00) |
| **Comparison with**  **MDS-UPDRS-III subitems for lower extremity** | **longer turning time** | all | 67 | 21 (31.34) | 28 (41.79) | 14 (20.90) | 0 (0.00) | 1 (1.49) | 3 (4.48) | 0 (0.00) |
|  |  | >\|mean + sd\| | 25 | 4 (16.00) | 13 (52.00) | 5 (20.00) | 0 (0.00) | 0 (0.00) | 3 (12.00) | 0 (0.00) |
|  | **more steps** | all | 67 | 22 (32.84) | 12 (17.91) | 11 (16.42) | 1 (1.49) | 1 (1.49) | 17 (25.37) | 2 (2.99) |

Note. Congruency and incongruency with our hypothesis are indicated with green and red shading respectively.

***Table 11.*** *Comparison turning and gait parameters (in* ***Freezers****)*

|  |  | n | **outer leg with shorter step length**  n (%) | **inner leg with shorter step length**  n (%) | **no asymmetry while turning**  n (%) | **missing value for turning**  n (%) |
| --- | --- | --- | --- | --- | --- | --- |
| **longer turning time** | all | 67 | 31 (46.27) | 33 (49.25) | 0 (0.00) | 3 (4.48) |
|  | >\|mean + sd\| | 32 | 12 (37.50) | 18 (56.25) | 0 (0.00) | 2 (6.25) |
| **more steps** | all | 67 | 19 (28.36) | 29 (43.28) | 0 (0.00) | 19 (28.36) |
|  | >\|mean + sd\| | 32 | 8 (25.00) | 18 (56.25) | 0 (0.00) | 6 (18.75) |

Note. Congruency and incongruency with our hypothesis are indicated with green and red shading respectively.
